# Nocturnal lighting in animal research should be replicable and reflect relevant ecological conditions

**DOI:** 10.1098/rsbl.2022.0035

**Published:** 2022-03-16

**Authors:** Anne E. Aulsebrook, Andreas Jechow, Annette Krop-Benesch, Christopher C. M. Kyba, Travis Longcore, Elizabeth K. Perkin, Roy H. A. van Grunsven

**Affiliations:** ^1^ Department of Behavioural Ecology and Evolutionary Genetics, Max Planck Institute for Ornithology, Seewiesen, Germany; ^2^ Community and Ecosystem Ecology, Leibniz Institute of Freshwater Ecology and Inland Fisheries, Berlin, Germany; ^3^ Remote Sensing and Geoinformatics, GFZ German Centre for Geosciences, Potsdam, Germany; ^4^ Initiative Nachhaltig Beleuchten, Berlin, Germany; ^5^ UCLA Institute of the Environment and Sustainability, Los Angeles, CA, USA; ^6^ Native Fish Society, Oregon City, OR, USA; ^7^ Dutch Butterfly Conservation, Mennonietenweg 10, 6702 AD, Wageningen, The Netherlands

**Keywords:** circadian, dim light, experimental design, light pollution, masking, melatonin

## Abstract

In nature, light is a key driver of animal behaviour and physiology. When studying captive or laboratory animals, researchers usually expose animals to a period of darkness, to mimic night. However, ‘darkness’ is often poorly quantified and its importance is generally underappreciated in animal research. Even small differences in nocturnal light conditions can influence biology. When light levels during the dark phase are not reported accurately, experiments can be impossible to replicate and compare. Furthermore, when nocturnal light levels are unrealistically dark or bright, the research is less ecologically relevant. Such issues are exacerbated by huge differences in the sensitivity of different light meters, which are not always described in study methods. We argue that nocturnal light levels need to be reported clearly and precisely, particularly in studies of animals housed indoors (e.g. ‘<0.03 lux’ rather than ‘0 lux’ or ‘dark’), and that these light levels should reflect conditions that the animal would experience in a natural context.

## 1. Introduction

In biological experiments, an organism's response to different treatments is typically compared to a control that represents conditions in a natural environment. The results of this comparison therefore depend not only on the effect of the treatment, but also on the control conditions. An important condition of most environments, which is frequently overlooked in current research, is the nocturnal light level. Exposing captive and laboratory animals to a 24 h cycle of darkness and light, to mimic night and day, might seem straightforward. However, the level of ‘darkness’ can be critical. ‘Darkness’ is a human concept with no quantitative definition, and even small differences in light levels can have large biological effects. When light levels are not reported adequately, experiments cannot be meaningfully replicated. Furthermore, when controls do not reflect natural conditions, the ecological relevance of research is questionable. Here, we aim to highlight the severity of this problem, and describe how to overcome it.

Light is a key driver of biological processes. Throughout evolutionary history, there have been predictable fluctuations in light, in relation to time of day, lunar phase and season [1]. Accordingly, almost all organisms have evolved daily, monthly and annual rhythms in their biology [1–3]. For example, animals typically have daily rhythms in sleep and metabolism, as well as seasonal rhythms in reproduction [1,3,4]. Light influences these biological rhythms primarily via melatonin, a photosensitive hormone that is highly conserved across taxonomic groups [5]. Light also facilitates animal vision, which in turn can facilitate behaviours such as spatial navigation, foraging and mating [4]. Given these diverse and pervasive effects on biology, light should be a fundamental consideration in all animal research. Nocturnal lighting is arguably particularly important, since light levels at night are a cue not only for time of day, but also lunar phase and daylength [1]. Nevertheless, there are two common issues with how nocturnal lighting is reported and used in animal research, which we outline below.

First, the descriptions of dark conditions in many published studies are inaccurate or imprecise. Light levels are most often reported in lux^1^, a unit that represents illuminance as perceived by the human eye under daytime adaptation. Many published studies, including recent studies, report nocturnal light conditions as ‘0 lux’ or simply as ‘dark’ (e.g. [6–9]). In a strictly physical sense, 0 lux means there are no photons, which is not physically possible in a standard laboratory setting. When a light meter detects ‘no light’, researchers should report that the light level was below the measurement capability of their instrument and specify what this limit was (e.g. ‘<0.03 lux’). This matters because the measuring capabilities of different instruments are vastly different. For example, the most common commercial light meters have a resolution of 1 lux, but a precision of several lux (i.e. a minimum sensitivity of 3 or more lux). From a visual perspective, a report of ‘0 lux’ could therefore represent anything from the conditions experienced several meters from a streetlight, to being unable to see at all.

**Figure 1.**
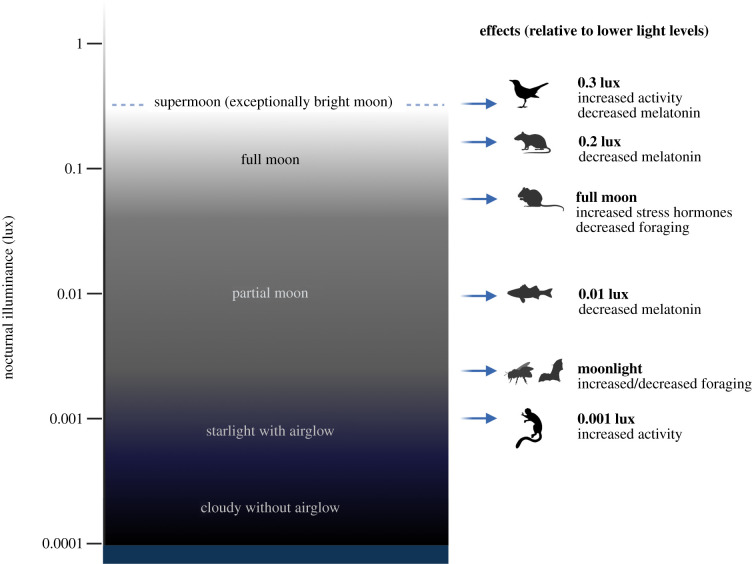
Nocturnal light levels below 1 lux or even 0.1 lux, which are often described simply as ‘dark’ in biological studies, can have substantial effects on animal behaviour and physiology. The left panel shows approximate nocturnal illuminance during various lunar phases and environmental conditions (cloudy without airglow = black, starlight with airglow = dark blue, partial moon = medium grey, full moon = pale grey, supermoon = dashed line) [11–13]. The right panel provides examples of biological effects that can occur at these illuminance levels, compared with lower light levels. These include (from bottom to top) increased activity in owl monkeys (*Aotus azarai*) [14], increased foraging in nocturnal bees (*Lasioglossum texanum*) [15], decreased foraging in fruit bats (*Cynopterus sphinx*) [16], decreased melatonin production in Eurasian perch (*Perca fluviatilis*) [17], increased cortisol and decreased foraging in spiny mice (*Acomys cahirinus* and *A. russatus*) [18], decreased plasma melatonin in nude rats (*Rattus norvegicus*) [19], and decreased plasma melatonin and increased activity in blackbirds (*Turdus merula*) [20]. Created with BioRender.com with additional illustrations from *PhyloPic* (blackbird by Anthony Caravaggi and owl monkey by E. Lear/Yan Wong).

Second, creating an extremely dark environment at night is not necessarily desirable or realistic [3,10]. Even in a natural setting, without artificial lights, most animals experience light at night from the moon, airglow and stars (figure 1). When there is very little light, such as during overcast skies when the moon is set, even nocturnal animals may become inactive at night. For example, nocturnal bees (*Lasioglossum texanum*) only forage at night after twilight when there is moonlight present [15]. Owl monkeys (*Aotus azarai*) also decrease their nocturnal activity during new moon nights and compensate by increasing their activity the following morning [14]. Very dark conditions in studies of captive animals could similarly mask natural nocturnal behaviour, forcing nocturnal animals to become active during light periods or extending inactive periods in diurnal or crepuscular animals. Thus, researchers might misinterpret the results of their research [3]. In some contexts, researchers might be led to underestimate the effects of the experimental treatment. In other contexts, abnormally dark conditions might amplify or even cause differences between the treatment and control. For example, a topic of growing interest is the effect of light pollution on wildlife. Light pollution, typically defined as increased light at night due to anthropogenic sources, can have pervasive effects on animal behaviour, physiology, reproduction and survival [21,22]. In studies of light pollution, researchers often compare a treatment of dim light at night (dLAN) to a dark control. Differences in behaviour between dLAN and dark control are then attributed to ‘negative’ impacts of light. However, if the control conditions are too dark, behaviours under dLAN might actually be normal nocturnal behaviours that the animal would exhibit in the wild, with behavioural differences being caused by unnaturally low light during the control.

Asking researchers to distinguish between very low light levels, in the range of 0.001 to 0.1 lux, may seem unnecessarily fastidious. However, these differences matter. A well-studied example is the influence of moonlight on animal behaviour [2]. Across the lunar cycle, illuminance can vary from <0.006 lux (starlight during a new moon or when the moon is set) to around 0.3 lux, with a summertime full moon typically in the range of 0.05 to 0.20 lux [11,12]. Even at such low light intensities, moonlight influences a wide range of behaviours, including coral spawning [23], foraging in nocturnal animals [15,16,24] and singing in diurnal songbirds [25]. Studies of light pollution now demonstrate these influences as well (e.g. [10,25,26]). Describing <0.1 lux as ‘darkness’ is therefore far too imprecise; there is still a wide range of biologically relevant light levels below this threshold.

Low intensities of light can also have important effects on physiology, including the suppression of melatonin [10,13]. Exposure to light, particularly blue wavelengths of light, suppresses melatonin production primarily through non-visual pathways [5,13]. Studies have found that light intensities as low as 0.028 lux (monochromatic blue light) and 0.3 lux (white light) are capable of suppressing melatonin in rodents and birds, respectively [13]. One recent study found that even 0.01 lux light at night reduces melatonin production in freshwater fish, compared to a ‘control’ below 0.00167 lux [17]. These examples underscore our point that results can be misleading if control conditions either too dark or too bright, and impossible to interpret when control conditions are not properly reported.

Studies of animals housed indoors should use an ecologically relevant level of light during the dark phase, which will depend on the animal and its natural habitat. Natural nocturnal light levels will be very different for a beetle in dense forest undergrowth, for example, compared with a bison on the open plains ([27]; also see below). Ideally, researchers should decide the most appropriate light level by measuring light in the animal's natural habitat, using a suitable device. This is more straightforward for some animals than others. Some research animals might come from heavily light-polluted environments, where light levels are increased above natural levels. Researchers must then decide whether to use these same light levels in the laboratory, or whether to mimic (hypothetical) natural light conditions in the absence of anthropogenic influences (based on the published literature, modelling or measurements in a different location). In such cases, the best approach will depend on the research question. Biomedical studies also often use inbred laboratory models, which can be quite different from their wild-type relatives. For these studies, environmental measurements may be less relevant, but the animal's physiology and visual capacity should still be taken into careful consideration. Previous research has already highlighted issues with the use of sub-thermoneutral housing temperatures in mouse studies [28]; suboptimal lighting has the potential to bias outcomes in a similar way.

In field studies, establishing ecologically relevant light conditions is rarely an issue, since animals are already being studied in their natural environment. Nevertheless, nocturnal light levels are still relevant for interpreting results, as well as comparing results between studies. This is true not only for nocturnal species, but also for diurnal animals whose sleep and subsequent daytime behaviour is influenced by light at night [4,29,30]. Nocturnal light conditions are influenced by many different features of an animal's habitat, including reflectance of the ground, shading from overhanging vegetation, shielding of light by rocks and mountains, attenuation by the atmosphere and water, and light pollution. Conditions also vary temporally, due to factors such as cloud cover, lunar phase, and season [12,31]. For example, ground reflectance may vary seasonally depending on snow cover, vegetation, water coverage (e.g. flooding) and moisture. These differences are not trivial; cloud cover combined with snow cover can increase suburban sky brightness by a factor of about 190 [31]. This variability in nocturnal light, from both anthropogenic and natural sources, is important to consider when designing and reporting study methods.

Importantly, controlling and measuring nocturnal lighting requires researchers to understand the precision of their measuring devices. It is therefore imperative for researchers to be wary of the issues with measuring light and to use devices with an appropriate level of precision for their research. For some studies, it may be helpful to seek help from researchers that deal more centrally with light (e.g. astrophysicists, optical physicists or visual ecologists). In many cases, commercial DSLR cameras may also be a suitable alternative to illuminance meters [11,32,33]. Researchers should also be careful to avoid inadvertently exposing research animals to unnecessary light; for example, light-emitting power buttons and monitors might need to be covered at night. Here, it is also important to consider that some animals can detect wavelengths of light that humans cannot, such as UV and infrared light [34,35].

Experimental studies rely on meaningful comparisons [3,36]. To draw reasonable conclusions, we require a clear understanding of experimental and natural conditions, and how these affect the study animal. Sometimes, the most basic aspects of an animal's environment can be the easiest to overlook. Nocturnal lighting is not the only environmental variable that researchers need to consider, but it is one that is frequently neglected in current research. Fortunately, the issue is also reasonably simple to address. As previously highlighted by the editors of *Nature Neuroscience*, ‘the key to reproducibility is accurate reporting of these seemingly mundane details, which potentially have large effects' [37]. We would also add that to interpret the results of animal research, we need to consider the perspective of the study animal: what they see, what they experience and how the research environment compares to their natural habitat.

## Data Availability

This article has no additional data.

## Footnotes

1 We are aware that the human-centric photometric quantity of illuminance might not be the most appropriate choice for all studies. However, this discussion is not focused on which metric to use, but rather on how to report metrics and design controls. We emphasize that the concepts discussed here also apply to other radiometric units, such as irradiance, and that reports of ‘0 W m^−2^’ or ‘0 photons s^−1^ m^−^^2^’ should also be avoided.
